# Strengthening infection prevention and control during the COVID-19 pandemic: implementation of a decentralised mentorship model across 450 primary health facilities in Sierra Leone, 2021–2022

**DOI:** 10.3389/frhs.2026.1732035

**Published:** 2026-02-23

**Authors:** Eric Nzirakaindi Ikoona, Fatima Tsiouris, Oliver Eleeza, Ronald R. Mutebi, Amon Njenga, AbdulRaheem Yakubu, Amy Elizabeth Barrera-Cancedda, Heather E. Fosburgh, Christiana Kallon, Miriam Rabkin, Mame Awa Toure, Susan Michaels-Strasser

**Affiliations:** 1 ICAP at Columbia University, Freetown, Sierra Leone; 2 ICAP at Columbia University, New York, NY, United States; 3 Department of Epidemiology, Columbia University Mailman School of Public Health, New York, NY, United States; 4 Resolve to Save Lives, Epidemic Ready Primary Health Care, New York, NY, United States; 5 Infection Prevention and Control, Ministry of Health, Freetown, Sierra Leone

**Keywords:** COVID-19, health care worker training, health system resilience, health system strengthening, infection prevention and control, mentorship, primary health care, Sierra Leone

## Abstract

**Background:**

The COVID-19 pandemic exposed significant infection prevention and control (IPC) gaps in Sierra Leone's primary health care system. We evaluated whether a decentralised multicomponent mentorship model could improve IPC performance across 450 government primary health facilities and support sustainable domestic financing for IPC.

**Methods:**

We conducted a pre-post quasi-experimental evaluation without a comparison group using facility-level indicators at baseline (April 2021) and endline (January 2022). The intervention package included competency-based IPC training, twice-monthly facility mentorship using structured observation checklists, routine monitoring with feedback using national IPC assessment tools configured in District Health Information Software 2 (DHIS2), targeted support for IPC commodities and water, sanitation and hygiene (WASH)-related infrastructure, community engagement, and budget advocacy through policy briefs and stakeholder meetings. We compared paired facility indicators using McNemar's chi-square test and examined service delivery patterns using month-matched comparisons to pre-pandemic levels (April 2019–January 2020). Primary outcome domains included training coverage, IPC supplies and infrastructure availability, and observed adherence to core IPC practices.

**Results:**

Facilities meeting ≥80% staff training coverage increased from 38% to 100% (*p* < 0.001). Availability of IPC SOPs/registers, triage infrastructure, and core IPC supplies improved in 20 of 22 indicators (*p* < 0.001). Observed adherence improved for hand hygiene (39% to 89%), appropriate mask use (50% to 98%), screening at entry (27% to 96%), waste segregation (21% to 98%), and sharps safety (89% to 100%) (all *p* < 0.001). Service delivery volumes were maintained or increased during the intervention period compared to pre-pandemic levels for six of eight indicators examined (*p* ≤ 0.007). The Ministry of Health (MoH) established the first dedicated IPC budget line, increasing domestic allocation by 25% from USD 384,000 to USD 480,000, with USD 2.3 million secured from partners.

**Conclusions:**

A decentralized mentorship model embedded in government structures can rapidly strengthen primary care IPC capacity while catalysing the policy and financing commitments essential for sustainability. The consistency of improvements across indicators supports this approach for similar low-resource settings. Controlled designs are needed to establish attribution and assess long-term impact.

## Introduction

Coronavirus disease (COVID-19) exposed major gaps in infection prevention and control (IPC) in low-resource health systems, increased risk for health care workers (HCWs), and disrupted essential services (1, 2). In Sierra Leone, the 2014–2016 Ebola epidemic had already revealed IPC weaknesses, claiming 221 HCW lives. These HCWs represented 6.2% of all Ebola deaths and 11% of Sierra Leone's physician workforce (3–5). Service use and public trust declined substantially during and after the outbreak (6, 7). Strong IPC in primary care protects patients, communities, and HCWs and supports continuity of care during health system shocks (8, 9). During COVID-19, workforce shortages, supply constraints, and delayed vaccine access further increased HCW exposure risk in Sierra Leone (10, 11).

Global and national guidance define core IPC requirements. These include a functioning programme with trained staff, multimodal strategies, routine monitoring with feedback, and water, sanitation and hygiene (WASH)-enabled infrastructure backed by dedicated financing (1, 5, 12). However, these guidelines often assume stable supervision capacity, reliable supply chains, and predictable financing, conditions that are difficult to sustain at scale in primary health care systems during public health emergencies. Yet assessments conducted during COVID-19 in Sierra Leone documented gaps in supplies, supervision, and adherence to protocols that constrained safe service delivery (8, 11).

Evidence from low- and middle-income countries demonstrates that training alone rarely sustains IPC practice change. Supportive supervision, mentorship, and audit and feedback mechanisms are essential complements (13, 14). Decentralised mentorship models, in which trained mentors conduct regular facility visits with structured observation and feedback, have shown promise for improving clinical practices including IPC in resource limited settings (15, 16). Such approaches align with World Health Organization (WHO) recommendations for multimodal IPC improvement strategies that combine education, monitoring, reminders, safety culture, and system change (5).

Strong IPC practices during health emergencies may help maintain patient and community confidence in facility safety and support continued health care-seeking behaviour (8, 9). Visible safety measures can reassure patients that facilities are safe. These include entry screening, staff masking, and hand hygiene availability. Such measures may mitigate service disruptions commonly observed during disease outbreaks (7, 17). On this basis, we hypothesised that IPC strengthening could be associated with maintenance of essential service utilisation during the COVID-19 period compared to pre-pandemic levels. We did this while recognising that multiple contextual factors influence service delivery during emergencies.

ICAP at Columbia University (ICAP) and Resolve to Save Lives (RTSL) launched a multi-phase, multi-country IPC initiative in 2020. The initiative delivered competency-based training to frontline HCWs in 11 African countries, including Sierra Leone (18). In Sierra Leone, ICAP and RTSL partnered with the Ministry of Health (MoH) to implement both Phase I and Phase II. Phase I emphasised rapid skill building across selected facilities. Phase II expanded this work by scaling a decentralised mentorship, monitoring, and advocacy model to 450 government primary health facilities across seven districts, embedding implementation within existing government structures**.**

The Phase II intervention package combined competency-based training with twice-monthly facility-level mentorship using national tools and structured checklists, routine monitoring with feedback through District Health Information Software 2 (DHIS2), targeted IPC commodity and WASH support, community engagement, and explicit budget advocacy using policy briefs and stakeholder meetings. As part of this strategy, the MoH and partners set a programme benchmark of at least 20% growth in domestic IPC national budget allocations to support sustainability beyond project funding. This benchmark served as an advocacy target aligned with WHO emphasis on dedicated IPC financing (5).

This paper evaluates whether a decentralised mentorship model implemented through government systems improved IPC training coverage, availability of IPC commodities and infrastructure, and adherence to IPC practices across 450 primary health facilities during the COVID-19 pandemic. We also describe systems-level outcomes, including strengthening and domestic budget advocacy results. Consistent with WHO guidance to monitor continuity of essential services during health emergencies, we include a descriptive analysis of routine DHIS2 service volumes during the intervention period compared with a month-matched pre-pandemic baseline (19).

## Methods

### Evaluation design and period

We conducted a pre-post quasi-experimental evaluation without a comparison group. The evaluation was embedded in an IPC strengthening intervention implemented through government structures. Implementation ran from 1 March 2021 to 31 March 2022 (Supplementary File S1: Implementation timeline). We collected facility-level IPC baseline measurements in April 2021 and the analytic endline measurements in January 2022. For descriptive service delivery patterns, we compared April 2019 to January 2020 (pre-COVID-19) with April 2021 to January 2022 (during COVID-19). We used month-matched facility-month pairs to reduce seasonality bias, consistent with WHO guidance to monitor continuity of essential health services during COVID-19 (19, 20).

### Setting and facilities

The intervention covered 450 primary health facilities across seven districts, representing approximately 35% of Sierra Leone's 1,286 primary health facilities, and serving an estimated 60% of the national population (4,228,111/7,092,113) (21).

### Facility selection criteria

MoH, district teams, and implementing partners selected seven districts and 450 government primary health facilities based on three criteria. First, they ensured geographic representation across Western Area, Northern, Southern, and Eastern regions. Second, they assessed operational feasibility for mentorship coverage. Third, they required routine DHIS2 reporting completeness of at least 80%. Facilities met inclusion criteria if they were active government primary health facilities with ongoing service delivery and routine reporting. Private facilities and tertiary hospitals were excluded. Selection was purposive rather than random. This maximised coverage and aligned with existing government systems.

### Intervention

The intervention package comprised six core activities. These aligned with WHO IPC core components and the Sierra Leone National IPC Guidelines (5, 12).

First, each facility designated an IPC focal person and assigned trained personnel for screening and triage (Core Component 1). Second, teams distributed and operationalised national IPC guidelines, cleaning and disinfection SOPs, and triage protocols (Core Component 2). Third, all HCWs received competency-based training on standard, droplet, and airborne precautions; Personal Protective Equipment (PPE) donning and doffing; hand hygiene; and waste management (Core Component 3). Fourth, mentors conducted twice-monthly facility visits combining on-the-job coaching with reminders, job aids, and infrastructure problem-solving (Core Component 4). Fifth, mentors used structured observation checklists, entered data into DHIS2, and conducted monthly performance reviews with facility teams (Core Component 5). Sixth, facilities established dedicated outdoor triage areas with natural ventilation, functional hand hygiene stations, waste management infrastructure, and core IPC commodities (Core Components 7 and 8). Training coverage targets of at least 80% of facility staff ensured adequate workforce capacity (Core Component 6).

### Adaptation for primary health facilities

We adapted WHO and national IPC guidance to a minimum feasible package for primary care, given baseline infrastructure constraints (5, 12). We prioritised high-yield outpatient practices: hand hygiene, entry screening and triage, appropriate mask use, and waste segregation (1, 5). We emphasised simple, risk-reducing infrastructure, including outdoor triage with natural ventilation and at least one metre spacing, plus functional hand hygiene stations at entry and care points (1, 12). Commodity support focused on core items for standard precautions, including medical masks, examination gloves, hand hygiene supplies, chlorine solutions, and labelled waste bins (1, 5). We deprioritised specialised PPE such as N95 respirators and face shields, consistent with risk-based PPE guidance that reserves respirators for aerosol-generating procedures and higher-risk contexts (1, 5). Community engagement supported minor repairs and compound cleaning when government resources were insufficient (12).

### Delivery model and staffing

**Figure 1:**
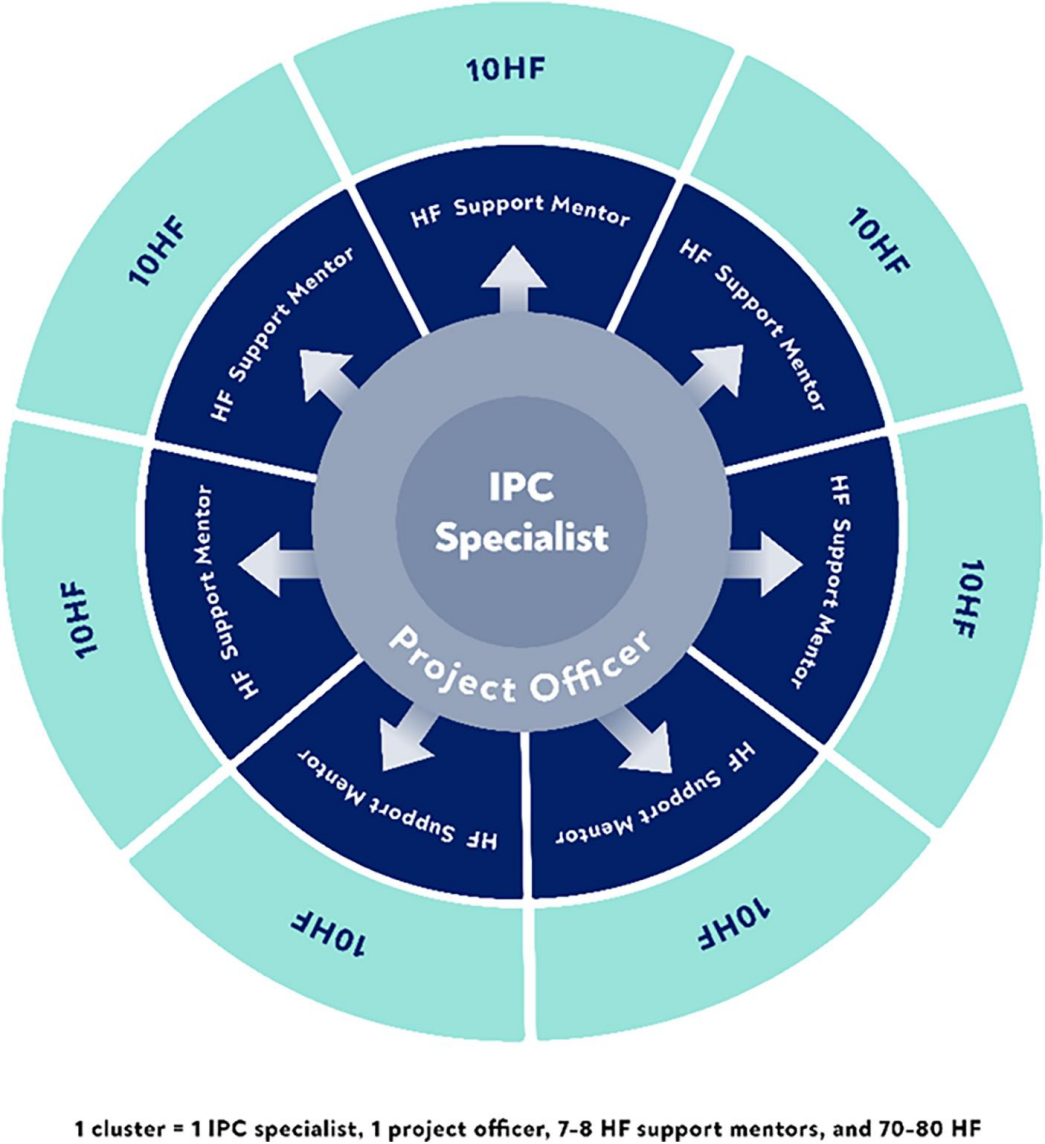
Cluster team structure and health facility coverage.

The project recruited 58 total staff members. These included 45 IPC mentors (HCWs with specialised IPC training), 6 project officers, 6 national MoH IPC specialists, and 1 monitoring and evaluation officer. The MoH IPC specialists provided technical oversight. Mentorship support was organised into six district “cluster teams.” Each team consisted of one project officer, one MoH IPC specialist, and 7–8 IPC mentors. A team covered 70–80 facilities. Each mentor supported approximately 10 primary health facilities (Figure 1). This structure yielded a 1:10 mentor-to-facility ratio.

### Training and mentorship component

All HCWs at intervention facilities received standardised orientation. Content covered standard, droplet, and airborne precautions. It also covered donning and doffing PPE. Training used national IPC guidelines and WHO recommendations. Training utilised competency-based approaches with structured curricula and post-training assessment tools (Supplementary File S2: Training curriculum outline).

Mentors conducted twice-monthly in-person facility visits (approximately every two weeks). At least monthly visits occurred when access was constrained by flooded roads during the rainy season. Between visits, mentors provided remote support via phone, SMS, WhatsApp, and Zoom to reinforce corrective actions and troubleshoot issues. Mentorship used structured observation checklists, on-the-job coaching, and feedback using national mentorship tools and job aids developed by MOH (Supplementary File S3: Mentorship observation checklist). Teams spread skills across staff rather than relying on single IPC focal persons. Visits were conducted in collaboration with District Health Management Teams (DHMTs).

### Performance monitoring and adaptive intensity

A standardised baseline IPC assessment using the Sierra Leone National IPC Assessment Tool in April 2021 identified gaps in training coverage, supplies, infrastructure, and practice (Supplementary File S4: Assessment tool and scoring rubric). The tool assessed 33 indicators across four domains: workforce and training (6 indicators), SOPs and guidelines (3 indicators), infrastructure (6 indicators), and supplies and equipment (18 indicators). Facilities received a composite score representing the percentage of indicators met and domain-specific scores.

Mentorship intensity was adapted based on baseline performance. Facilities scoring below 50% received twice-monthly visits with extended duration of four to six hours. Facilities scoring 50%–75% received twice-monthly standard visits of two to three hours. Facilities scoring above 75% received monthly visits with lighter-touch follow-up. Teams reviewed monthly results with facility leadership and DHMTs. They agreed on corrective actions using national thresholds (12).

### Measurement of IPC practice adherence

Mentors assessed IPC practices during routine facility workflows. They used a structured national observation checklist with standardised indicator definitions (Supplementary File S5: Indicator definitions and data dictionary). This included tool use, indicator definitions, and supervised practice observations before facility visits.

During mentorship visits, mentors conducted structured observations in high-flow clinical areas and triage points. For hand hygiene, mentors recorded observed hand hygiene opportunities as the denominator. They recorded correct technique at the appropriate WHO Five Moments as the numerator. For mask use, mentors recorded the number of HCWs observed in clinical areas as the denominator. They recorded the number wearing a medical mask correctly covering nose and mouth as the numerator. For entry screening, mentors assessed screening practices using triage registers and direct observation. They recorded patients presenting as the denominator. They recorded patients screened for COVID-19 symptoms as the numerator. For waste segregation and sharps management, mentors assessed performance through direct inspection against national standards. These were binary indicators at facility level (met or not met).

### Quality assurance

Project officers independently verified a sample of 10% of facility visit assessments. They reconciled discrepancies with mentors and facility leadership during monthly coordination meetings. We did not calculate formal inter-rater reliability metrics. We report this as a limitation.

### IPC commodities and infrastructure

Mentors worked with DHMTs and facility teams to quantify consumption and forecast IPC supply needs. To close immediate gaps, the project supplied selected commodities. These included surgical masks, examination gloves, impermeable aprons, detergent, 0.5% chlorine for blood and body fluid spills, 0.1% chlorine for surfaces and floors, buckets, labelled waste bins, infrared thermometers, and hand hygiene stations. Facilities established dedicated, preferably outdoor triage areas with at least one metre spacing and natural ventilation, per national guidance (12).

### Community engagement

Facility managers mobilised community members for compound cleaning, minor repairs, and improved waste management.

### Systems strengthening and budget advocacy

With MoH, the project revitalised the national IPC Technical Working Group. The project supported the group to clarify their terms of reference and to develop a national IPC roadmap. The group helped synthesise routine programme and DHIS2 data into four policy briefs. These briefs were released in June 2021, September 2021, November 2021, and January 2022. Findings were presented at two breakfast meetings.

In September 2021, attendees included MoH Director of Planning and Policy, the Director of Primary Health Care, IPC Program Manager, DHMT representatives from seven districts, and partner organisations including CDC, WHO, and UNICEF. In December 2021, attendees included MoH leadership, Ministry of Finance budget officers, and donor representatives.

Policy briefs presented routine performance data demonstrating IPC improvements. They made the investment case for sustained domestic IPC financing. They targeted at least 20% growth in domestic allocations.

### Outcomes and definitions

Definitions followed national guidelines and WHO recommendations (5, 12).

### Primary outcomes

IPC mentorship coverage (facility-level, binary) included the following indicators at least 80% of HCWs trained in the last 6 months on standard, droplet, airborne precautions and donning and doffing PPE; presence of a dedicated IPC focal person; trained personnel assigned to screening and triage.

IPC supplies, equipment, and infrastructure (facility-level, binary) included availability and use of SOPs and registers; infrastructure indicators (ventilation, dedicated entry screening and triage, outdoor triage separate from patient care, at least one metre spacing, functional hand hygiene stations with supplies in triage and patient-care areas); and supplies indicators (infrared thermometer, masks, gloves, N95 respirators, impermeable aprons, detergent, buckets, water, 0.5% chlorine, labelled waste bins, 0.1% chlorine, face shields and goggles, elbow-length gloves).

IPC practice adherence (facility-level performance) included correct hand hygiene (WHO “Five Moments”), appropriate mask use by HCWs in clinical areas, entrance screening for COVID-19 symptoms, waste segregation from source to disposal and or treatment, and correct management of sharps containers (emptied at three-quarters full) (12, 22).

### Secondary outcomes

Essential-service utilisation (facility-month counts) included antenatal care visits; TB cases diagnosed (GeneXpert or smear); persons initiating ART; malaria diagnostic tests performed; measles-containing vaccine doses administered; patients with diagnosed hypertension; HIV tests among pregnant women; and sputum specimens collected for TB.

### Tertiary outcomes

Budget advocacy outcomes included number and content of policy briefs, attendance and decisions at breakfast meetings, and MoH budget documents evidencing dedicated IPC budget lines and domestic allocation increases vs. at least 20% target.

### Data sources and management

Mentors collected programme data on password protected tablets. They used standardised national tools configured to the MoH managed DHIS2 platform. Monthly IPC summaries were validated with facility in-charges and DHMT focal persons. A monitoring and evaluation officer checked completeness and flagged outliers. The officer reconciled discrepancies with mentors. We analysed only aggregate, non-identifiable data. We abstracted data on IPC budget allocation from official MoH budget and financial reports.

### Statistical analysis

For paired facility-level binary indicators (training, supplies and infrastructure, practice; Tables 1–3), we used McNemar's chi-squared test to compare April 2021 with January 2022 (two-sided alpha = 0.05). We used Wilson method to calculate confidence intervals for proportions.

**Table 1:** Percentage difference between health facility with trained health care workers in IPC at baseline and endline, 450 primary health facilities, Sierra Leone.

#	Indicators	Baseline performance April 2021% (*n*)	Endline performance January 2022% (*n*)	Difference in performance % 95% CI	*p*-value
1	Percentage of HF with ≥80% of HCWs trained on standard precautions within the last 6 months (*N* = 450)	38 (172)	100 (450)	62 (58-66)	<0.001
2	Percentage of HF with ≥80% of HCWs trained on the topics within the last 6 months on Droplet precautions (*N* = 450)	38 (171)	100 (450)	62 (58–66)	<0.001
3	Percentage of HF with ≥80% of HCWs trained on the topics within the last 6 months on Airborne precautions (*N* = 450)	38 (173)	100 (450)	62 (58–66)	<0.001
4	Percentage of HF with ≥80% of HCWs trained on the topics within the last 6 months on donning and doffing PPE (*N* = 450)	38 (172)	100 (450)	62 (58–66)	<0.001
5	Percentage of HF with a dedicated IPC professional or person with IPC training (*N* = 450)	64 (290)	100 (450)	36 (32–40)	<0.001
6	Percentage of HF with dedicated and trained personnel on screening and triage (*N* = 450)	38 (170)	100 (450)	62 (58–66)	<0.001

CI, confidence interval.

**Table 2:** IPC resources availability and use at baseline and endline, 450 primary health facilities in seven districts, Sierra Leone, 2022.

#	Indicators	Baseline performance April 2021% (*n*)	Endline performance January 2022% (*n*)	Difference in performance % (95% CI)	*p*-value
Availability and use of guidelines, SOPs and registers					
1	Percentage of HF having a guideline or SOP available on how to perform cleaning and disinfection (*N* = 450)	25 (111)	100 (450)	75 (71–79)	<0.001
2	Percentage of HF having a cleaning schedule that has been filled out daily (*N* = 450)	12 (54)	100 (450)	88 (85–91)	<0.001
3	Percentage of HF with triage forms and registers available and properly utilised (*N* = 450)	8 (35)	100 (450)	92 (89–95)	<0.001
	IPC infrastructure				
4	Percentage of HF with natural ventilation in all patient care and waiting areas (*N* = 450)	85 (383)	99 (447)	14 (11–17)	<0.001
5	% of HF with dedicated screening and triage area for each open entry point (*N* = 450)	34 (153)	99 (447)	65 (61–69)	<0.001
6	% of HF with dedicated outdoor screening and triage areas, separate from patient care areas (*N* = 450)	34 (151)	98 (441)	64 (59–69)	<0.001
7	% of HF with dedicated screening and triage areas with at least 1 m separation between patients (*N* = 450)	34 (151)	99 (446)	65 (59–69)	<0.001
8	% of functional hand hygiene stations in screening and triage areas with adequate supplies (Baseline *N* = 534, Endline *N* = 610)	71 (380)	99 (604)	28 (24–32)	<0.001
9	% of functional hand hygiene stations in patient care areas with adequate supplies (Baseline *N* = 1,223, Endline *N* = 1,367)	83 (1,018)	99 (1,358)	16 (14–19)	<0.001
	IPC equipment and supplies				
10	% of HF with functional infrared no-touch thermometer in the screening and triage area being used (*N* = 450)	43 (195)	100 (450)	57 (52–62)	<0.001
11	% of HF with surgical face mask readily available (*N* = 450)	55 (246)	98 (440)	43 (38–48)	<0.001
12	% of HF with examination gloves readily available (*N* = 450)	69 (310)	91 (410)	22 (17–27)	<0.001
13	% of HF with N95 respirators readily available (*N* = 450)	20 (91)	35 (157)	15 (9–21)	<0.001
14	% of HF with impermeable apron (*N* = 450)	42 (189)	89 (401)	47 (42–52)	<0.001
15	% of HF with detergent (*N* = 450)	49 (222)	100 (450)	51 (48–54)	<0.001
16	% of HF with at least two buckets (*N* = 450)	69 (312)	100 (450)	31 (27–35)	<0.001
17	% of HF with water (*N* = 450)	70 (315)	99 (444)	29 (25–33)	<0.001
18	% of HF with 0.5% chlorine solution for disinfection of blood and body fluid spills (*N* = 450)	25 (114)	100 (450)	75 (71–79)	<0.001
19	% of HF with covered, sealed, and labelled (infectious and non-infectious) waste bins available at all patient service points (*N* = 450)	20 (92)	100 (450)	80 (76–84)	<0.001
20	% of HF with 0.1% chlorine solution for disinfection of surfaces and floors (*N* = 450)	21 (94)	98 (441)	77 (73–81)	<0.001
21	% of HF with face shields/goggles readily available (*N* = 450)	46 (208)	48 (216)	2(-5–9)	0·32
22	% of HF with elbow-length gloves readily available (*N* = 450)	39(175)	40(180)	1(−5- 7)	0·393

CI, confidence interval.

Items 8–9 use hand hygiene stations denominators and not primary health facility denominator.

**Table 3:** IPC practice standards at baseline and endline, 450 primary health facilities in seven districts, Sierra Leone, 2022.

#	Indicators	Baseline performance April 2021% (*n*)	Endline performance January 2022% (*n*)	% Difference (95% CI)	*p*-value
1	% of HF with sharps containers available at all points of use that are emptied when three-quarters full (line or tape should demark the ¾ mark) (*N* = 450)	89 (399)	100 (450)	11 (8–14)	<0.001
2	% of HF with waste sorted (e.g., indicated by colours or labelling) according to the type of waste from source, during collection, to disposal and/or treatment (*N* = 450)	21 (95)	98 (443)	77 (73–81)	<0.001
3	% of patients screened at the entrance to HF for COVID-19 symptoms (Baseline *N* = 147,211; Endline *N* = 180,123)	27 (40,408)	96 (173,051)	69 (68–69)	<0.001
4	% of observed correct hand hygiene practice (Baseline *N* = 165, Endline *N* = 2,201)	39 (64)	89 (1,963)	50 (42–58)	<0.001
5	% of HCWs who are always wearing medical/surgical masks appropriately when they work in clinical areas (Baseline *N* = 928, Endline *N* = 1,314)	50 (460)	98 (1,287)	48 (45–51)	<0.001

CI, confidence interval.

For essential-service utilisation (Table 4), we assessed normality of month-matched facility-month counts using the Shapiro–Wilk test. Indicators with normally distributed differences were analysed using paired t-tests (antenatal care, TB diagnoses, ART initiation, malaria tests, measles vaccination, and hypertension diagnoses). Wilcoxon signed-rank tests were applied for non-normal indicators (HIV testing during pregnancy and TB sputum collection).

**Table 4:** Analysis of the number of individuals accessing eight select essential health services pre-COVID-19 and during COVID-19 at 450 primary health facilities in Sierra Leone.

#	Indicators	Pre-COVID-19 mean visits (SD)	During COVID-19 mean visits (SD)	Mean difference (95% CI)	T-test statistic	d.f.	*p*-value
1	Number of antenatal care visits	57.74 (65.48)	60.59 (70.26)	2.86 (1.02–4.70)	3.048	487	0.002
2	Number of TB cases diagnosed by GeneXpert or smear	0.4 (4.46)	0.52 (5.08)	0.12 (0.02–0.22)	2.347	486	0.019
3	Number of persons with HIV started on antiretroviral therapy	1.72 (18.01)	2.2 (27.41)	0.48 (0.13–0.83)	2.716	487	0.007
4	Number of patients on whom diagnostic tests for malaria were performed	146.56 (128.55)	158.03 (146.36)	11.46 (6.89–16.02)	4.923	488	<0.001
5	Number of doses of measles-containing vaccine administered	21.51 (20.91)	26.48 (23.70)	4.97 (4.35–5.59)	15.695	487	<0.001
6	Number of patients seen with a diagnosis of hypertension	1.94 (5.88)	2.89 (6.28)	0.96 (0.76–1.16)	9.329	487	<0.001
7	Number of pregnant women tested for HIV †	54.98 (1,904.95)	16.42 (23.05)	-38.60 (−94.33 to 7.14)	−1.358	484	0.175
8	Number of patients from whom sputum was collected for diagnostic testing for TB	2.44 (23.44)	2.37 (22.73)	−0.08 (−0.34–0.23)	−0.500	488	0.617

Pre-COVID-19, April 2019 to January 2020; During COVID-19, April 2021 to January 2022; SD, standard deviation; df, degrees of freedom; CI, confidence interval; HIV, human immunodeficiency virus; TB, tuberculosis.

Analysed using Wilcoxon signed-rank test due to non-normal distribution; median differences not calculated for non-parametric tests. Large SDs reflect facility-level variation in service volumes, with some facilities serving substantially larger catchment populations than others.

The 450 facilities represented 35% of Sierra Leone's primary health facilities. They were selected to ensure broad geographic representation across seven districts and integration with existing DHIS2 systems. This sample provided greater than 80% power to detect a 20-percentage point change in binary outcomes at alpha = 0.05, assuming a baseline prevalence of 40% for key IPC indicators (based on preliminary assessments) and accounting for the paired facility design.

Given the exploratory nature of secondary outcomes, we did not adjust for multiple comparisons (23). All comparisons reflect within-group changes from baseline to endline, since the intervention was delivered universally. Analyses were conducted using Stata 16.0 (24).

## Results

### Healthcare workers trained on IPC

Table 1 shows that all the six training-related indicators increased from baseline to endline. Facilities with ≥80% of staff trained on standard, droplet, and airborne precautions and on donning/doffing PPE rose from 38% to 100%. Facilities with an IPC focal person rose from 64% to 100% (all *p* < 0.001)

### IPC supplies, standard operating procedures, and infrastructure

Table 2 highlights statistically significant improvement in all three indicators related to the availability and use of guidelines, standard operating procedures (SOPs), and registers, as well as in all six indicators related to the availability of IPC infrastructure (*p* < 0.001). Of the 13 equipment/supplies indicators, 11 improved (*p* < 0.001); face shields/goggles and elbow-length gloves showed no significant change.

### IPC practice

Table 3 shows that all five practice indicators improved: correct hand hygiene, appropriate HCW mask use, entry screening, waste segregation, and sharps safety (all *p* < 0.001)

### Continuity of essential services

Table 4 shows that six of eight essential service indicators increased between the pre-COVID-19 period (April 2019–January 2020) and the COVID-19 period (April 2021–January 2022): antenatal care visits, malaria diagnostic tests, measles vaccination, hypertension diagnoses, TB diagnoses, and ART initiation (all *p* ≤ 0.019). HIV testing in pregnancy and TB sputum collection showed no significant change (*p* > 0.05)

### Systems strengthening and advocacy outcomes

Four policy briefs were presented at two breakfast meetings with MoH leadership and partners. The MoH introduced IPC Budget Code 22040301 in FY2022—the first dedicated IPC line item in Sierra Leone's national health budget. Domestic IPC allocation increased by 25% from USD 384,000 to USD 480,000. Partners committed USD 2.3 million in complementary support.

## Discussion

### Principal findings

This evaluation of a large-scale IPC strengthening package implemented through government structures across 450 primary health facilities showed substantial gains in three primary domains. Training coverage, availability of IPC supplies and infrastructure, and observed adherence to core IPC practices all improved significantly from baseline to endline. The intervention period also coincided with systems changes that could have been associated with it. These governance and financing outcomes align with expectations in global IPC guidance (5). The findings support a decentralised, competency-based mentorship model embedded in routine government systems as a feasible approach to strengthen IPC capacity during health emergencies.

Achieving at least 80% IPC training coverage in all supported facilities from a baseline of 38% represents a substantial national-scale shift. More consequential are the large practice effects. Correct hand hygiene increased from 39% to 89%. Appropriate mask use increased from 50% to 98%. Patient screening at entrances increased from 27% to 96%. Waste segregation increased from 21% to 98%. Evidence indicates that training alone rarely sustains behaviour change. Iterative, on-site mentorship with observation, feedback, and problem-solving improves adherence (15, 16, 25). The package combined competency-based education with twice-monthly facility-based mentorship using national checklists and job aids. This operationalised multimodal improvement recommended by WHO and national guidance (5, 12). Alignment between rapid gains in hand hygiene stations and large improvements in hand hygiene practice matches evidence that infrastructure access enables adherence (22, 26).

Marked improvements in SOPs, registers, functional hand hygiene stations, triage areas, and core consumables indicate that teams addressed both “know-how” and “can-do” constraints. Cleaning SOPs and schedules reached near-universal availability by endline. Facilities instituted outdoor, well-ventilated triage with at least one metre spacing, reflecting national recommendations and pragmatic adaptation (12).

Face shields/goggles and elbow-length gloves did not improve significantly. This reflected deliberate budget choices to prioritise high-yield commodities for primary care IPC. This finding also highlights that resource improvements partially depended on direct project procurement rather than strengthened local supply systems. The lack of improvement in these specialised items reflects persistent budget trade-offs and supply chain limitations for more expensive PPE in resource-constrained primary care settings. Prioritisation of essential over specialised items is defensible in primary care contexts when risk assessments guide escalation for aerosol-generating procedures (5, 8).

Very low baseline performance in comprehensive waste management and improvement to 98% underscore how targeted coaching, labelled bins, and steady chlorine supply close large gaps quickly, which matters given occupational and environmental risks of poor waste handling (27, 28).

Descriptive analysis showed that service delivery was maintained or increased during the intervention period for most indicators examined. WHO recommends monitoring essential service delivery during COVID-19 to detect indirect effects and guide mitigation strategies (19). Six of eight tracer indicators showed higher volumes during April 2021 to January 2022 compared with the month-matched pre-pandemic baseline. These included increases in TB diagnoses and ART initiation. This pattern contrasts with service disruptions documented in many settings (2, 17).

However, these findings require cautious interpretation. Multiple factors likely influenced service delivery patterns beyond IPC improvements. These could be changes in national COVID-19 policies, partner support for service delivery, community care seeking and avoidance related to perceived risk, commodity availability, and health system adaptation over time (19, 29, 30). The pre-post design without comparison facilities cannot disentangle these influences. We did not measure patient perceptions. Evidence from Ebola in Sierra Leone indicates that perceived facility risk and visible IPC measures can influence facility use (31). The service delivery analysis provides context on health system functioning during the intervention. It does not establish that IPC improvements caused service continuity. Future research using comparison groups or interrupted time series designs can assess causal pathways between IPC strengthening and service utilisation.

Beyond facility-level change, the intervention reactivated the national IPC Technical Working Group and supported the development of a national IPC roadmap. Policy briefs using routine data were presented in high-level fora. These activities were associated with inclusion of a dedicated IPC budget line and increased domestic allocations. The introduction of IPC Budget Code 22040301 represents the first dedicated IPC line item in Sierra Leone's national health budget. Dedicated programme lines and predictable funding are core elements of sustainable IPC programmes (5). Initial disbursement represents progress toward institutionalisation.

The integration of budget advocacy with technical support distinguishes this model from IPC quality-improvement efforts that end when project financing ceases. In this programme, policy briefs and stakeholder meetings used routine performance and cost information to support the case for a dedicated IPC budget line and increased domestic allocation. This approach offers a feasible pathway for linking facility-level improvement to national financing decisions.

### Implementation insights for replication

Three delivery features appear central to effect and scalability. First, the cluster-team structure with a 1:10 mentor-to-facility ratio enabled regular on-site presence and remote follow-up. This maintained short feedback cycles despite access constraints. Second, adaptive intensity allowed targeting of lower-performing facilities with more frequent contacts while providing lighter oversight elsewhere. Third, supply-chain coaching paired with mentorship addressed bottlenecks that undermine behaviour change. However, direct commodity provision was necessary for rapid improvements (15). Community mobilisation for facility improvements complemented these efforts and supported local ownership (32).

### Strengths and limitations

This evaluation has several strengths. The large scale (450 primary health facilities across seven districts, representing 35% of all primary care facilities in Sierra Leone) enhances statistical power and external validity. The use of standardised national MoH IPC assessment tools strengthens data reliability and enables replication across routine government systems. Integration with the existing DHIS2 platform improved data quality and completeness. A prespecified, month-matched analytic design helped control for seasonal variation (20).

The pre-post design without a comparison group limits causal inference (33). National COVID-19 guidance in Sierra Leone evolved during the intervention period, including adaptations to case management protocols, HCW quarantine procedures, and infection prevention requirements (34). Partner support for essential health services continued through parallel programmes (8). Community care-seeking behaviour during disease outbreaks is influenced by perceived facility risk and visible infection control measures, as documented during Ebola in Sierra Leone (7, 31). These concurrent factors may have contributed to observed improvements independently of the intervention. The large effect sizes, consistency across multiple indicators, and alignment with program theory support plausibility of intervention effects (33). However, definitive causal attribution is not possible with this design. Future evaluations should consider stepped-wedge or comparison-group designs where feasible and ethical (35).

Facilities were selected purposively for geographic distribution and DHIS2 readiness rather than through random sampling. This may limit generalisability beyond similar contexts. We did not stratify results by district, facility type, or urban/rural setting. This limits assessment of equity in gains. Improvements in IPC supply availability reflected direct project support, including provision of selected commodities. These should not be interpreted as evidence of long-term supply chain sustainability. Future evaluations should distinguish between government-procured vs. externally supplied commodities to better assess national procurement system resilience.

Mentor-observed adherence to IPC practices may have been influenced by Hawthorne effects (staff performing better during observations). Repeated observations, large effect sizes, and structured tools help mitigate but do not eliminate this bias. The lack of formal inter-rater reliability assessment limits confidence in measurement consistency across mentors. Mentor-observed facilities that mentors themselves supported may inflate performance estimates.

The evaluation focused on IPC process indicators rather than health care-associated infections or transmission rates. Sierra Leone's primary care system lacks routine surveillance for such outcomes. Process measures are recognised proxies for IPC performance (5).

Finally, the evaluation did not include cost or cost-effectiveness analysis. This limits the ability to assess resource efficiency. Sustainability post-2022 remains unclear pending continued government budget disbursement and transition from project commodity supply to government procurement.

### Implications

For Sierra Leone and similar contexts, a model implemented through government structures that couples competency-based supportive supervision with performance monitoring, WASH and supply enablement, community mobilisation, and explicit budget advocacy can deliver meaningful IPC gains. Several actions are critical for sustainability.

Protect and expand the IPC budget line: Advocate for sustained or increased domestic IPC allocation in successive budget cycles. Link routine IPC indicator data to budget justifications.
Embed mentorship in district structures: Transition project-supported mentors to government payroll or integrate mentorship functions into existing DHMT supervision frameworks.
Transition commodity supply: Shift from direct project provision to strengthened government and partner procurement systems to avoid creating supply dependencies.
Define minimum IPC standards: Establish a minimum, risk-based IPC commodity and WASH package for primary facilities that can be sustained through routine procurement.
Link IPC to supervision cycles: Integrate IPC indicators into routine facility supervision and performance review frameworks.

Future work should include cost and cost-effectiveness analyses. Where feasible, tracking of health care-associated and occupational infection outcomes would strengthen the investment case (36).

## Conclusion

This evaluation demonstrates that a decentralised mentorship model embedded in government structures can rapidly strengthen primary care IPC capacity at scale. This approach also catalysed the policy and financing commitments essential for sustainability. In Sierra Leone, targeted budget advocacy resulted in the first dedicated IPC budget line in the national health budget. This achievement offers a pathway for translating program evidence into durable system change. The consistency of improvements across training, infrastructure, and practice indicators supports applicability in similar low-resource settings. Future research should employ controlled designs to establish causal attribution. Cost-effectiveness analysis and assessment of whether gains persist beyond implementation are also needed.

## Data Availability

The raw data supporting the conclusions of this article will be made available by the authors, without undue reservation.

## Supplementary material

Supplementary File S1 Implementation timeline.

Supplementary File S2 Training curriculum outline and competency assessment form.

Supplementary File S3 Mentorship structured observation checklist.

Supplementary File S4 Baseline IPC assessment tool and scoring rubric.

Supplementary File S5 Indicator definitions and data dictionary.
