# Supplementary material for: Strengthening infection prevention and control during the COVID-19 pandemic: implementation of a decentralised mentorship model across 450 primary health facilities in Sierra Leone, 2021–2022
Source: Front Health Serv. 2026 Feb 23;6:1732035. doi: 10.3389/frhs.2026.1732035 (PMC12968192; doi:10.3389/frhs.2026.1732035)
Supplement: Supplementary file 1 [file Table1.docx]

| **SUPPLEMENTARY FILE 1: Implementation Timeline** | | | | | | | | | | | | | | |
| --- | --- | --- | --- | --- | --- | --- | --- | --- | --- | --- | --- | --- | --- | --- |
| *IPC Strengthening in 450 Primary Health Facilities, Sierra Leone (February 2021 - January 2022)* | | | | | | | | | | | | | | |
|  |  |  | **2021** | | | | | | | | | | | **2022** |
| **#** | **Activity** | **Output/Indicator** | **Feb** | **Mar** | **Apr** | **May** | **Jun** | **Jul** | **Aug** | **Sep** | **Oct** | **Nov** | **Dec** | **Jan** |
| 1 | Hire ICAP program staff | 58 staff hired and oriented | **●** |  |  |  |  |  |  |  |  |  |  |  |
| 2 | Engage with key IPC national stakeholders | National IPC TWG revitalized | **●** |  |  |  |  |  |  |  |  |  |  |  |
| 3 | Conduct baseline IPC assessment at all 450 facilities | Gaps identified across 450 HFs | **●** | **●** |  |  |  |  |  |  |  |  |  |  |
| 4 | Procure and distribute IPC commodities and supplies | 450 HFs received IPC supplies | **●** | **●** |  |  |  |  |  |  |  |  |  |  |
| 5 | Develop mentorship plan with DHMTs | 6 cluster teams established | **●** | **●** |  |  |  |  |  |  |  |  |  |  |
| 6 | Train IPC mentors and specialists (6-day program) | 45 mentors + 6 specialists trained |  | **●** | **●** |  |  |  |  |  |  |  |  |  |
| 7 | Conduct competency-based IPC training for HCWs | ≥80% HCWs trained per facility |  | **●** | **●** |  |  |  |  |  |  |  |  |  |
| 8 | Configure national IPC tools in DHIS2 | IPC dashboard operational |  | **●** | **●** |  |  |  |  |  |  |  |  |  |
| 9 | Implement twice-monthly mentorship visits | ≥85% facilities visited monthly |  |  |  | **●** | **●** | **●** | **●** | **●** | **●** | **●** | **●** | **●** |
| 10 | Collect routine IPC indicators via DHIS2 | ≥95% facilities reporting |  |  |  | **●** | **●** | **●** | **●** | **●** | **●** | **●** | **●** | **●** |
| 11 | Conduct quarterly cluster review meetings | 3 review meetings conducted |  |  |  |  |  | **●** |  |  | **●** |  |  | **●** |
| 12 | Develop and implement QI plans for low-performing sites | QI plans monitored monthly |  |  |  | **●** | **●** | **●** | **●** | **●** | **●** | **●** | **●** | **●** |
| 13 | Produce and present policy briefs | 4 briefs, 2 breakfast meetings |  |  |  |  |  | **●** |  |  | **●** |  | **●** | **●** |
| 14 | Conduct endline assessment | Endline data collected |  |  |  |  |  |  |  |  |  |  |  | **●** |
| 15 | Final report and dissemination | Report submitted |  |  |  |  |  |  |  |  |  |  |  | **●** |
|  |  |  |  |  |  |  |  |  |  |  |  |  |  |  |

**SUPPLEMENTARY FILE 2**

**Training Curriculum Outline and Competency Assessment Form**

*Strengthening Infection Prevention and Control During the COVID-19 Pandemic:*

*Implementation of a Decentralized Mentorship Model Across*

*450 Primary Health Facilities in Sierra Leone*

ICAP at Columbia University | Resolve to Save Lives | Ministry of Health, Sierra Leone

**2021-2022**

**PART A: TRAINING CURRICULUM OUTLINE (6-Day Program)**

| **DAY** | **TOPICS AND ACTIVITIES** |
| --- | --- |
| **1** | **Foundations:** COVID-19/EVD epidemiology, risk factors, case definitions, symptomology, IPC principles overview |
| **2** | **Standard & Transmission-Based Precautions:** Hand hygiene (soap/water, ABHR), WHO 5 Moments scenarios, PPE donning/doffing. Tools: Hand washing checklists, WHO observation tool |
| **3** | **Operational IPC:** Screening/triage, waste management, environmental cleaning, injection safety, community guidance, indicator definitions, data flow |
| **4** | **Field Practicum:** Health facility assessment visits, data collection practice, gap identification, multimodal improvement strategies, findings presentation |
| **5** | **Quality Improvement:** QI standards, methodology introduction, QI tools practice, change ideas and PDSA cycles |
| **6** | **Data Systems & Assessment:** Tablet/DHIS2 training, data collection piloting, post-test competency assessment, training evaluation |

**PART B: COMPETENCY ASSESSMENT FORM**

*Competency threshold: 80% (10/12 correct required)*

| Name: _________________________ | Date: _________________________ |
| --- | --- |

**1. Hand washing does not need to be done by HCWs if using gloves.**

a) True b) False

**2. Standard precautions protect:**

a) HCWs only b) Patients only c) Visitors only d) All of the above

**3. Source segregation of waste into colour coded bags is the first step of clinical waste management.**

a) True b) False

**4. When discarding a needle and syringe after injection:**

a) Separate and discard separately b) Discard as one unit c) Discard needle, reuse syringe d) Leave in locker

**5. Select the INCORRECT statement about breaking disease transmission:**

a) Practice hand hygiene b) Use aseptic technique c) Give all patients antibiotics d) Cover skin breaks

**6. Disinfecting an item means it is sterile and ready for invasive procedures.**

a) True b) False

**7. The mode of transmission of COVID-19 is:**

a) Airborne and Contact b) Droplets and Contact c) Droplets only d) Airborne only

**8. Transmission-based precautions may be used with Standard Precautions.**

a) True b) False

**9. Quality Assurance measures performance at a single point in time.**

a) True b) False

**10. The know-do gap refers to HCWs doing what they know they should not.**

a) True b) False

**11. Only QI officers can implement QI projects.**

a) True b) False

**12. Which is NOT part of the QI project lifecycle?**

a) Form QI team b) Identify challenge c) Assign blame d) Develop change ideas

| Score: ___/12 Result: ☐ Competent (≥10) ☐ Requires retraining (<10) Assessor: ____________ |
| --- |

Answer Key: 1.b 2.d 3.a 4.b 5.c 6.b 7.b 8.a 9.a 10.b 11.b 12.c

**SUPPLEMENTARY FILE 3**

**Mentorship Structured Observation Checklist**

*Strengthening Infection Prevention and Control During the COVID-19 Pandemic:*

*Implementation of a Decentralized Mentorship Model Across*

*450 Primary Health Facilities in Sierra Leone*

ICAP at Columbia University | Resolve to Save Lives | Ministry of Health, Sierra Leone

**2021-2022**

**FACILITY INFORMATION**

| Health Facility Name: _________________________ | Date of Visit: _________________________ |
| --- | --- |
| District: _________________________ | Chiefdom: _________________________ |
| Mentor Name: _________________________ | Visit Number: ☐ 1st ☐ 2nd ☐ Follow-up |

**SECTION A: HAND HYGIENE OBSERVATION**

| **Indicator** | **Yes** | **No** | **N/A** |
| --- | --- | --- | --- |
| A1. Functional hand hygiene station at facility entrance | ☐ | ☐ | ☐ |
| A2. Hand hygiene station in patient care area(s) | ☐ | ☐ | ☐ |
| A3. Water available at hand hygiene stations | ☐ | ☐ | ☐ |
| A4. Soap or ABHR available at hand hygiene stations | ☐ | ☐ | ☐ |
| A5. HCW observed performing hand hygiene at appropriate moment | ☐ | ☐ | ☐ |
| A6. Correct hand hygiene technique observed (all steps) | ☐ | ☐ | ☐ |

**Hand Hygiene Score: ___/6**

**SECTION B: PERSONAL PROTECTIVE EQUIPMENT (PPE)**

| **Indicator** | **Yes** | **No** | **N/A** |
| --- | --- | --- | --- |
| B1. Medical masks available for HCWs | ☐ | ☐ | ☐ |
| B2. HCWs in clinical areas wearing masks correctly (covering nose and mouth) | ☐ | ☐ | ☐ |
| B3. Examination gloves available | ☐ | ☐ | ☐ |
| B4. Impermeable aprons/gowns available | ☐ | ☐ | ☐ |
| B5. PPE donning/doffing poster visible | ☐ | ☐ | ☐ |

**PPE Score: ___/5**

**SECTION C: SCREENING AND TRIAGE**

| **Indicator** | **Yes** | **No** | **N/A** |
| --- | --- | --- | --- |
| C1. Designated screening/triage area at entrance | ☐ | ☐ | ☐ |
| C2. Triage area outdoors or well-ventilated | ☐ | ☐ | ☐ |
| C3. At least 1 meter spacing in waiting area | ☐ | ☐ | ☐ |
| C4. Infrared thermometer available and functional | ☐ | ☐ | ☐ |
| C5. Patients screened for COVID-19 symptoms at entry | ☐ | ☐ | ☐ |
| C6. Screening register available and in use | ☐ | ☐ | ☐ |

**Screening/Triage Score: ___/6**

**SECTION D: WASTE MANAGEMENT**

| **Indicator** | **Yes** | **No** | **N/A** |
| --- | --- | --- | --- |
| D1. Color-coded waste bins available (infectious/general) | ☐ | ☐ | ☐ |
| D2. Waste bins labeled and lined with appropriate bags | ☐ | ☐ | ☐ |
| D3. Sharps container available in clinical areas | ☐ | ☐ | ☐ |
| D4. Sharps container not more than 3/4 full | ☐ | ☐ | ☐ |
| D5. Waste segregated correctly at point of generation | ☐ | ☐ | ☐ |
| D6. Safe final disposal method in place (pit/incinerator) | ☐ | ☐ | ☐ |

**Waste Management Score: ___/6**

**SECTION E: ENVIRONMENTAL CLEANING**

| **Indicator** | **Yes** | **No** | **N/A** |
| --- | --- | --- | --- |
| E1. Detergent available for routine cleaning | ☐ | ☐ | ☐ |
| E2. 0.1% chlorine solution available for surfaces | ☐ | ☐ | ☐ |
| E3. 0.5% chlorine solution available for blood/body fluids | ☐ | ☐ | ☐ |
| E4. Cleaning schedule displayed | ☐ | ☐ | ☐ |
| E5. Clinical areas visibly clean | ☐ | ☐ | ☐ |

**Environmental Cleaning Score: ___/5**

**SUMMARY AND ACTION PLAN**

| **Domain** | **Score** | **Max** | **Priority Actions** |
| --- | --- | --- | --- |
| A. Hand Hygiene |  | 6 |  |
| B. PPE |  | 5 |  |
| C. Screening/Triage |  | 6 |  |
| D. Waste Management |  | 6 |  |
| E. Environmental Cleaning |  | 5 |  |
| TOTAL |  | 28 |  |

Overall Performance: ☐ <50% (Intensive support) ☐ 50-75% (Standard support) ☐ >75% (Light-touch)

Mentor Signature: ____________________ Facility In-Charge Signature: ____________________

Next Visit Date: ____________________

**SUPPLEMENTARY FILE 4**

**COVID-19 Checklist for Primary Care Facilities**

| N**ame of Health Facility:**  **Type of facility CHC [ ] MCHP [ ] CHP [ ]**  **Type of ownership Government [ ] Private [ ] Faith-Based [ ] NGOs [ ]** | | | | | | | |
| --- | --- | --- | --- | --- | --- | --- | --- |
| **Name of person conducting assessment**  **_________________________________**  **Telephone Contact: _________________________**  **Designation: _______________________** | | **Name of interviewee**  **____________________________________**  **Telephone Contact: ____________________________**  **Designation: _________________________** | | | | | |
| **Name of incharge (Name & contact)**  **__________________________________________**  **Role: (please select one)**  IPC Focal Person  **Incharge** Other | | **Number of Health Care Workers^^[[1]](#footnote-1)^^ Employed by Facility:** | | | | | |
| **District:** | | **Chiefdom:** | | | **City/Town/Village:** | | |
| **Date of Visit:** | | **Date of Last Visit:** | | | | | |
| **Surveillance** | | | | | | **Comments** | |
| 1a. | Total number of **patients seen** at this health facility in the previous month | | # | | |  | |
| 1b. | Number of **patients screened** on entrance to this health facility for COVID-19 symptoms in the previous month | | # | | |  | |
| 1c. | Number of **suspected COVID-19 patients** reported to local surveillance personnel from this health facility in the previous month | | # | | |  | |
| 2a. | Number of unique^^[[2]](#footnote-2)^^ health care workers **(HCWs)**Error! Bookmark not defined. **who reported to work** at this health facility in the previous month | | # | | |  | |
| 2b. | Number of new **suspected COVID-19 infections among HCWs** at this health facility in the previous month | | # | | |  | |
| 2c. | Number of new **confirmed COVID-19 infections among HCWs** at this health facility in the previous month | | # | | |  | |
| 2d. | Does the facility have an assessment and management protocol in place (including a register, assessment tools, communication, etc.) for exposed or confirmed HCWs? | | Yes | No | |  | |
| **Screening and Triage** | | | | | | **Comments** | |
| 3a. | Are dedicated screening and triage personnel trained and in place? | | Yes | No | |  | |
| 3b. | Does the health facility have a dedicated screening and triage area for each open entry point into the health facility? | | Yes | No | |  | |
| 3c. | Is/are the dedicated screening and triage area(s) outdoors and separated from patient care areas? | | Yes  No  N/A if 3b No | | |  | |
| 3d. | Does/do the dedicated screening and triage area(s) have adequate spacing between patients (2m preferred, but a minimum of 1m separation)? | | Yes  No  N/A if 3b No | | |  | |
| 3e. | Is a functional infrared no-touch thermometer available^^[[3]](#footnote-3)^^ in the screening and triage area and is it being used as per thermometer instructions^^[[4]](#footnote-4)^^? | | Yes | No | |  | |
| 3f. | Are triage forms and registers available^3^ and properly utilized? | | Yes | No | |  | |
| 3g. | Does the facility have a protocol for separation and isolation of patients with suspected COVID-19? | | Yes | No | |  | |
| **Infection Prevention and Control** | | | | | | **Comments** | |
| 4. | Does the facility have personnel trained in IPC/an IPC professional employed who is responsible and accountable for IPC program at the health facility? | | Yes | No | |  | |
| 5. | Does the health facility have a register/electronic database containing the names of the health workers who were trained, the training date, the training type, and the organization that provided the training? | | Yes | No | |  | |
| 6. | Have ≥80% of HCW at this facility been trained on the topics below within the last 6 months:    Standard precautions  Airborne precautions  Droplet precautions  Contact precautions  Donning and doffing PPE | | Yes  Yes  Yes  Yes  Yes | No  No  No  No  No | |  | |
| 7. | Does the facility have IPC guidelines or standard operating procedures (SOPs) | | Yes | No | |  | |
| 8. | Does the facility have IPC guidelines or standard operating procedures (SOPs) that are readily accessible to health facility staff? | | Yes | No | |  | |
| 9. | Is natural ventilation (windows not obstructed and able to be opened at all times) available in all patient care areas and waiting areas? | | Yes | No | |  | |
| **Hand Hygiene** | | | | | | **Comments** | |
| 10. | Total number of hand hygiene stations in the screening and triage areas (denominator) | | #  Please enter “N/A” if 3b is “No” | | |  | |
|  | Number of hand hygiene stations in the screening and triage areas that are functional and have adequate^^[[5]](#footnote-5)^^ supplies for use on the date of visit (numerator) | | #  Please enter “N/A” if 3b is “No” | | |  | |
| 11. | Total number of hand hygiene stations in patient care areas inside the health facility (denominator) | | # | | |  | |
|  | Number of hand hygiene stations in patient care areas inside the health facility that are functional and have adequate^5^ supplies for use on date of visit (numerator) | | # | | |  | |
| 12. | Total number of opportunities^^[[6]](#footnote-6)^^ observed where hand hygiene should have been performed (denominator) | | # | | |  | |
|  | Number of hand hygiene opportunities observed to be performed correctly (numerator) | | # | | |  | |
| **PPE** | | | | | | **Comments** | |
| 13. | Minimum stock of PPE available at the time of visit (see attachment for the minimum list of PPE for certain period) | | Yes  No | | |  | |
| 14. | All of the following PPE supplies are readily available for use   - Elbow gloves - Examination gloves - Face shields/goggles - Plastic apron - Surgical face mask - Gowns - Respirators/N-95 - No PPE Stock | | Yes  No  Yes  No  Yes  No  Yes  No  Yes  No  Yes  No  Yes  No  Yes  No  Yes  No | | | Stock out for past days  1–7 [ ] 8-14 [ ] 15-30 [ ] 30+ []  1–7 [ ] 8-14 [ ] 15-30 [ ] 30+ []  1–7 [ ] 8-14 [ ] 15-30 [ ] 30+ []  1–7 [ ] 8-14 [ ] 15-30 [ ] 30+ []  1–7 [ ] 8-14 [ ] 15-30 [ ] 30+ []  1–7 [ ] 8-14 [ ] 15-30 [ ] 30+ []  1–7 [ ] 8-14 [ ] 15-30 [ ] 30+ []  1–7 [ ] 8-14 [ ] 15-30 [ ] 30+ [] | |
| 15. | Report, request and issue voucher (RRIV) completed and archived chronologically | | Yes  No | | |  | |
| 16. | PPE supplies are stored in a safe location off the floor and in a dry place | | Yes  No | | |  | |
| 17. | There is a stock ordering book in use at the facility | | Yes  No | | |  | |
| 18. | Health facility is performing an inventory and ordering IPC supplies (based on the gap) at least once a month | | Yes  No | | |  | |
| 19. | Total number of HCWs who should have been wearing medical/surgical masks at all times when they worked in clinical areas during the observation period (denominator) | | # | | |  | |
|  | Number of HCWs who are wearing medical/surgical masks appropriately at all times when they work in clinical areas during the observation period (numerator) | | # | | |  | |
| 20. | Total number of HCWs who should have been wearing face shields or goggles at all times when they worked in clinical areas during the observation period (denominator) | | # | | |  | |
|  | Number of HCWs who are wearing face shields or goggles appropriately at all times when they work in clinical areas during the observation period (numerator) | | # | | |  | |
| **Environmental Cleaning and Waste Management** | | | | | | **Comments** | |
| 21a. | Is a guideline or SOP on how to perform cleaning and disinfection available? | | Yes | No | |  | |
| 21b. | Is there a cleaning schedule that has been filled out daily? | | Yes | No | |  | |
| 21c. | The environment is visibly clean (both inside and outside the facility, no litter scattered on grounds, no spills seen on the floors) | | Yes | No | |  | |
| 21d. | Have staff performing cleaning and disinfection been trained on cleaning, disinfecting, and health facility waste management? | | Yes | No | |  | |
| 21e. | Do cleaning staff clean with appropriate protective gear? (Must be observed)   - Eye protection (goggles and/or face shields) - Medical/surgical mask - Closed toe shoes or boots - Rubber gloves - Impermeable apron | | Yes  Yes  Yes  Yes  Yes | No  No  No  No  No | |  | |
| 21f. | Are required cleaning supplies available? (Must be observed)   - Water - At least two buckets: one for clean water and one for soapy water - Mop (1 per area) - Color-coded cleaning cloths - Cleaning solution (detergent) | | Yes  Yes  Yes  Yes  Yes | No  No  No  No  No | |  | |
| 21g | Is 0.5% chlorine solution available for disinfection of blood and body fluid spills? | | Yes | No | |  | |
| 21h. | Is 0.1% chlorine solution available for disinfection of surfaces and floors? | | Yes | No | |  | |
| 21i. | Please select **only** one of the following:  There is an accessible record of cleaning for ALL areas including floors, horizontal work surfaces, sinks, veronica buckets, reusable medical equipment, etc. which is completed and signed by the cleaners each day | |  | | |  | |
|  | There is an accessible record, but it is not completed and signed daily OR it is outdated | |  | | |  | |
|  | There is no record of areas including floors, horizontal work surfaces, sinks, veronica buckets, reusable medical equipment, etc. being cleaned | |  | | |  | |
| 22a. | The waste management SOP is available (check for physical copy of SOP) | | Yes | No | |  | |
| 22b. | There is appropriate bin liner in each bin (according to the color coding policy) | | Yes | No | |  | |
| 22c | Is waste sorted (e.g., indicated by colors or labeling) according to the type of waste: infectious, non-infectious, sharps (from source, during collection, to disposal (collection for removal from the HCF) and/or treatment – incineration or open pit burning))? | | Yes | No | |  | |
| 22d. | Are covered, sealed, and labelled (infectious and non-infectious) waste bins available at all patient service points? | | Yes | No | |  | |
| 22e. | There are no overfull waste bins in the wards (clinical area) | | Yes | No | |  | |
| 22f. | Waste segregation posters are clearly placed above all waste bins | | Yes | No | |  | |
| 22g. | All infectious or sharps waste is disposed inthe facilities | | Yes | No | |  | |
| 22h. | Verify existence of safe burning pit at the facility (fenced, brick enclosure at the ground, waste residues/ ashes buried) | | Yes | No | |  | |
| 22i. | There is a functional placenta pit | | Yes | No | |  | |
| 22j. | Are sharps containers available at all points of use? | | Yes | No | |  | |
| 22k. | Syringes & Needles are discarded in sharp box after single use | | Yes | No | |  | |
| 22l. | There is sharp injury report for PEP management | | Yes | No | |  | |
| 22n. | Sharp posters are clearly displaced where sharps are used/ prepared | | Yes | No | |  | |
| 22o. | There are no sharps on the floor (i.e. needles, scalpels, ampoules, etc) | | Yes | No | |  | |
| 22p. | The sharps containers are not filled above the fill mark (not above ¾) | | Yes | No | |  | |
| **Decontamination of Medical Equipment** | | |  |  | |  | |
| 23a. | There is SOP on cleaning, disinfection and/ or sterilization of surgical instruments and other medical devices (check for written SOP) | | Yes | No | |  | |
| 23b. | There is a designed area/ room for cleaning and disinfection of medical devices at the decontamination area | | Yes | No | |  | |
| 23c. | There is a functional autoclave in use to sterilize critical medical devices (e.g forecasts, scissors) | | Yes | No | |  | |
| 23d. | The sterilize packs are stored in a well-ventilated room or cabinet | | Yes | No | |  | |
| **Essential Services Continuity** | | |  |  | |  |  |
| **Antenatal Services** | | |  |  | |  |  |
|  |  | | **Past Month (2021)** | **Same Month 2019** | | **% Change** | **Comments** |
| 24. | Number of antenatal care visits in the past month & the same month in 2019 | |  |  | |  |  |
| 25. | Number of pregnant women tested for HIV in the past month & the same month in 2019 | |  |  | |  |  |
| **Tuberculosis** | | | | | | | |
| 26. | Number of patients from whom sputum was collected for diagnostic testing for TB in the past month and the same month in 2019 | |  |  | |  |  |
| 27. | Number of TB cases diagnosed by GeneXpert or smear in the past month and the same month in 2019 | |  |  | |  |  |
| **HIV** | | |  |  | |  |  |
| 28. | Number of persons with HIV started on anti-retroviral therapy (ART) in the past month and the same month in 2019 | |  |  | |  |  |
| **Malaria** | | |  |  | |  |  |
| 29. | Number of patients on whom diagnostic tests for malaria were performed in the past month (smear and rapid diagnostics) and in the same month in 2019 | |  |  | |  |  |
| **Vaccinations** | | |  |  | |  |  |
| 30. | Number of doses of measles-containing vaccine administered in the past month and in the same month in 2019 | |  |  | |  |  |
| **Cardiovascular Health** | | |  |  | |  |  |
| 31. | Number of patients with a diagnosis of hypertension seen in the past month and in the same month in 2019 | |  |  | |  |  |
| IPC Action plan | | |  |  | |  |  |
| 32. | An IPC action plan was developed based on previous assessment feedback | | Yes | No | |  | |
| 33. | The action plan has been implemented (see report for activity progress) | | Yes | No | |  | |

**SUPPLEMENTARY FILE 5: Indicator Definitions and Data Dictionary**

*IPC Strengthening in 450 Primary Health Facilities, Sierra Leone 2021-2022*

**DOMAIN 1: WORKFORCE AND TRAINING (6 indicators)**

| **Indicator** | **Definition** | **Numerator / Denominator** | **Data Source** | **Frequency** |
| --- | --- | --- | --- | --- |
| T1. Training coverage | Proportion of HCWs who completed IPC training in past 6 months | # HCWs trained / Total # HCWs at facility | Training register, HR records | Quarterly |
| T2. IPC focal person | Facility has designated IPC focal person with documented role | Binary: Yes/No | Facility assessment | Baseline/Endline |
| T3. Triage personnel | Trained staff assigned to screening/triage | Binary: Yes/No | Duty roster, observation | Monthly |
| T4. Standard precautions | Staff trained on standard precautions (hand hygiene, PPE) | # trained / Total HCWs | Training records | Quarterly |
| T5. Transmission-based | Staff trained on droplet/airborne precautions | # trained / Total HCWs | Training records | Quarterly |
| T6. PPE competency | Staff demonstrating correct donning/doffing | # competent / # assessed | Skills assessment | Quarterly |

**DOMAIN 2: SOPs AND GUIDELINES (3 indicators)**

| **Indicator** | **Definition** | **Numerator / Denominator** | **Data Source** | **Frequency** |
| --- | --- | --- | --- | --- |
| S1. IPC guidelines | National IPC guidelines available at facility | Binary: Yes/No | Document review | Baseline/Endline |
| S2. Cleaning SOPs | Environmental cleaning SOPs available and displayed | Binary: Yes/No | Observation | Monthly |
| S3. Triage protocol | COVID-19 screening protocol available at triage | Binary: Yes/No | Observation | Monthly |

**DOMAIN 3: INFRASTRUCTURE (6 indicators)**

| **Indicator** | **Definition** | **Numerator / Denominator** | **Data Source** | **Frequency** |
| --- | --- | --- | --- | --- |
| I1. Triage area | Dedicated screening/triage area at facility entrance | Binary: Yes/No | Observation | Monthly |
| I2. Outdoor/ventilated | Triage area outdoors or with adequate natural ventilation | Binary: Yes/No | Observation | Monthly |
| I3. Physical distancing | Waiting area allows ≥1 meter spacing between patients | Binary: Yes/No | Observation | Monthly |
| I4. HH station-entry | Functional hand hygiene station at facility entrance | Binary: Yes/No | Observation | Monthly |
| I5. HH station-care | Hand hygiene station in patient care area(s) | Binary: Yes/No | Observation | Monthly |
| I6. Water supply | Reliable water supply for hand hygiene and cleaning | Binary: Yes/No | Observation | Monthly |

**DOMAIN 4: SUPPLIES AND EQUIPMENT (18 indicators)**

| **Indicator** | **Definition** | **Numerator / Denominator** | **Data Source** | **Frequency** |
| --- | --- | --- | --- | --- |
| E1. Medical masks | Medical/surgical masks available (≥1 week stock) | Binary: Yes/No | Stock count | Monthly |
| E2. Exam gloves | Examination gloves available (≥1 week stock) | Binary: Yes/No | Stock count | Monthly |
| E3. N95/respirators | N95 or equivalent respirators available | Binary: Yes/No | Stock count | Monthly |
| E4. Aprons | Impermeable aprons/gowns available | Binary: Yes/No | Stock count | Monthly |
| E5. Eye protection | Face shields or goggles available | Binary: Yes/No | Stock count | Monthly |
| E6. Thermometer | Infrared thermometer available and functional | Binary: Yes/No | Equipment check | Monthly |
| E7. Soap | Soap available at hand hygiene stations | Binary: Yes/No | Observation | Monthly |
| E8. ABHR | Alcohol-based hand rub available | Binary: Yes/No | Observation | Monthly |
| E9. Detergent | Detergent available for routine cleaning | Binary: Yes/No | Stock count | Monthly |
| E10. Chlorine 0.1% | 0.1% chlorine solution for surface disinfection | Binary: Yes/No | Observation | Monthly |
| E11. Chlorine 0.5% | 0.5% chlorine for blood/body fluid spills | Binary: Yes/No | Observation | Monthly |
| E12. Buckets | Buckets available for cleaning solutions | Binary: Yes/No | Observation | Monthly |
| E13. Waste bins | Color-coded waste bins (infectious/general) | Binary: Yes/No | Observation | Monthly |
| E14. Sharps containers | Puncture-resistant sharps containers available | Binary: Yes/No | Observation | Monthly |
| E15. Bin liners | Appropriate bin liners/bags available | Binary: Yes/No | Observation | Monthly |
| E16. Elbow gloves | Elbow-length gloves for waste handling | Binary: Yes/No | Stock count | Monthly |
| E17. Screening register | COVID-19 screening register available | Binary: Yes/No | Document review | Monthly |
| E18. IPC register | IPC monitoring register in use | Binary: Yes/No | Document review | Monthly |

**IPC PRACTICE ADHERENCE INDICATORS (5 indicators)**

| **Indicator** | **Definition** | **Numerator / Denominator** | **Data Source** | **Frequency** |
| --- | --- | --- | --- | --- |
| P1. Hand hygiene | HCWs performing hand hygiene at WHO 5 Moments with correct technique | # correct HH / # HH opportunities observed | Direct observation | Monthly |
| P2. Mask use | HCWs in clinical areas wearing mask correctly (nose and mouth covered) | # correct mask / # HCWs observed | Direct observation | Monthly |
| P3. Entry screening | Patients screened for COVID-19 symptoms at facility entry | # patients screened / # patients presenting | Register, observation | Monthly |
| P4. Waste segregation | Waste correctly segregated at point of generation | Binary: Met/Not met per facility | Direct observation | Monthly |
| P5. Sharps safety | Sharps containers <3/4 full and managed correctly | Binary: Met/Not met per facility | Direct observation | Monthly |

**Scoring: Composite IPC score = (# indicators met / 33 total indicators) × 100%**

Performance thresholds: <50% = Intensive support required; 50-75% = Standard support; >75% = Light-touch follow-up

1. “Health workers are all people engaged in work actions whose primary intent is to improve health, including doctors, nurses, lab technicians, cleaning staff, drivers, social workers, and administrators, among others.”

   [WHO Charter – Health worker safety: a priority for patient safety](https://www.who.int/docs/default-source/world-patient-safety-day/health-worker-safety-charter-wpsd-17-september-2020-3-1.pdf?sfvrsn=2cb6752d_2). Students and volunteers should also be included. [↑](#footnote-ref-1)
2. Health care workers reporting to work more than once during the reporting period should only be counted once. [↑](#footnote-ref-2)
3. Available means the item is present at the healthy facility and easily accessible to all health care workers. [↑](#footnote-ref-3)
4. Maximum number of hours of use per day, rest period between patients, etc. [↑](#footnote-ref-4)
5. Adequate supplies: water, soap, and a functioning tap **AND/OR** alcohol-based hand rub [↑](#footnote-ref-5)
6. Hand hygiene opportunity is defined as any opportunity when hand hygiene should be performed (e.g., before and after examining a patient, before donning PPE, after doffing PPE, etc.). https://www.who.int/gpsc/tools/5momentsHandHygiene_A3.pdf?ua=1 [↑](#footnote-ref-6)
